# Herlyn-Werner-Wunderlich Syndrome: Comparison of Two Cases

**DOI:** 10.3390/ijerph17197173

**Published:** 2020-09-30

**Authors:** Mateusz Kozłowski, Katarzyna Nowak, Dominika Boboryko, Sebastian Kwiatkowski, Aneta Cymbaluk-Płoska

**Affiliations:** 1 Department of Gynecological Surgery and Gynecological Oncology of Adults and Adolescents, Pomeranian Medical University, 70-111 Szczecin, Poland; kn13222@gmail.com (K.N.); dominikaboboryko@gmail.com (D.B.); aneta.cymbaluk@gmail.com (A.C.-P.); 2 Department of Obstetrics and Gynecology, Pomeranian Medical University, 70-111 Szczecin, Poland; kwiatkowskiseba@gmail.com

**Keywords:** HWWS, Herlyn-Werner-Wunderlich Syndrome, obstructed hemivagina and ipsilateral renal anomaly (OHVIRA), uterus didelphys, renal agenesis, congenital malformation, 3D ultrasound

## Abstract

Background: Herlyn-Werner-Wunderlich Syndrome is a rare malformation syndrome characterized by uterus didelphys with obstructed hemivagina and ipsilateral renal agenesis. Symptoms appear most often after menarche and are secondary to hematocolpos. We compare clinical symptoms, diagnosis and treatment of two patients, a 13-year-old and a 17-year-old. Case report: Despite the non-uniform clinical symptoms, it should be noted that in both patients, the 13-year-old and the 17-year-old, hematocolpos, which was probably the cause of lower abdominal pain, was diagnosed with ultrasound. The diagnosis was complemented by laparoscopy, which determined the diagnosis of malformation of uterus didelphys with obstructed hemivagina. The patients had a history of kidney agenesis, which, after gynecological diagnosis, turned out to be ipsilateral. In the 13-year-old, agenesis was diagnosed by uroscintigraphy, while in the 17-year-old it was diagnosed by urography. Incision and drainage of the residual vagina was performed in the course of therapeutic management. In both cases, the clinical situation required a repeated widening of the orifice. Conclusions: Lower abdominal pain accompanying hematocolpos suggested Herlyn-Werner-Wunderlich Syndrome (HWWS) as the cause of symptoms. 3D transvaginal ultrasound enabled the determination of a congenital uterine defect with high probability, although inconclusive cases required confirmation by laparoscopy. Incision of the blocked vagina and drainage of hematocolpos were the key components of treatment. The treatment of HWWS is a multi-step process.

## 1. Introduction

Herlyn-Werner-Wunderlich Syndrome (HWWS) is a rare congenital malformation syndrome characterized by a triad of uterus didelphys with obstructed hemivagina and ipsilateral renal agenesis [1], also known as OHVIRA [2]. OHVIRA stands for obstructed hemivagina and ipsilateral renal anomaly [3]. The prevalence of obstructive Müllerian malformations is unknown, but is reported to be 0.1% to 3.8% in women [4,5]. The prevalence of Herlyn-Werner-Wunderlich Syndrome is unknown. Literature data are ambiguous. Tuna et al. state that 6% of patients with uterine duplication have an obstructed hemivagina, and that renal agenesis is found in 63–81% of uterine duplications and in 92–100% of those with obstructed hemivagina [6]. According to Nishu et al., the frequency is 0.1–3% [3], while Fachin et al. state that it is 5% [7]. The lack of unambiguous information may be due to the fact that there is a lack of common terminology, and that could be the reason that it is reported as rare. Moreover, it should be remembered that in some cases the disease is asymptomatic during childhood and puberty. The diagnosis only occurs in adulthood when a woman is diagnosed with infertility and the cause is looked for. At 8–12 weeks of fetal life, caudal parts of the Müllerian ducts merge, resulting in the formation of a primary double-cavity uterus. In the 20th week of pregnancy, the septum regresses, and a single-cavity uterus is formed [8]. Therefore, arrested development of the Müllerian and mesonephric ducts at 8 weeks of gestation can result in an anomaly, such as uterus didelphys [9]. Clinical manifestations are unspecific and most often include abdominal pain, painful menstruation and a palpable mass in the abdomen, secondary to hematocolpos [10]. Symptoms most often appear during puberty, although in cases of incompletely obstructed hemivagina, the onset of symptoms may occur at a later time [11]. Ultrasound, magnetic resonance imaging and laparoscopy are used to establish the diagnosis. Therapeutic management consists of the drainage of hematocolpos—a pelvic tumor and the cause of clinical symptoms. Proper diagnosis and timely treatment are essential to avoid complications, including infertility. We present two cases of HWWS in a 13-year-old and a 17-year-old patient. We compare their symptoms, diagnostic methods and treatment.

## 2. Case Reports

Both patients were referred to the Department of Gynecological Surgery and Gynecological Oncology of Adults and Adolescents at the Pomeranian Medical University of Szczecin (hereinafter referred to as the Department) for diagnosis and specialist treatment. Both were pre-diagnosed in regional centers.

The 17-year-old patient presented with severe lower abdominal pain, which caused her to report to the district hospital. The patient menstruated from the age of 13; menstruation was regular and painful. In the hospital, gynecological examination revealed an uneven, painful, left-sided mass, 10 × 5 cm in diameter. In a speculum examination, a 3 mm hole in the left vaginal vault was demonstrated and yellowish exudate was evacuated. The exudate was collected for microbiological examination, which demonstrated the growth of numerous Staphylococcus epidermidis and a few Escherichia coli. After antibiotic therapy (cefuroxime, metronidazole), the palpable mass was reduced in size to 2 × 3 cm, the pain subsided and purulent content ceased to drain out of the hole in the vaginal vault. An ultrasound examination of the abdomen was performed twice, failing to visualize the left kidney. Moreover, the right kidney showed characteristics of compensatory hypertrophy. The absence of the left kidney was confirmed by urography. During hospitalization at the Department, a transvaginal ultrasound examination revealed a split uterine body with two cavities and a tumor measuring 71 × 32 mm on the left side. The uterine cavity was also dilated to 8 mm. The presence of a double uterine body was confirmed during a diagnostic laparoscopy, which also demonstrated unaffected appendages. In the course of therapy, the fluid collection in the blocked vagina was drained transvaginally, evacuating bloody, purulent content. Resection of the left hemivagina was performed and antibiotic therapy was implemented. No perioperative or postoperative complications occurred. Subsequent follow-up hospitalizations took place during menstruations. The clinical condition required two procedures to widen the opening of the left vagina. They were performed under general anesthesia during two consecutive hospitalizations. During subsequent hospitalizations, the left vagina was found to be patent. Eventually, the size of the fluid reservoir was significantly reduced to 40 × 33 mm as per the ultrasound examination. In addition to surgery, the patient also received oral estrogen-progestogen therapy. Based on the investigations, the patient was diagnosed with uterus didelphys with obstructed left hemivagina, left renal agenesis, pyohematocolpos and pyohematometra. She underwent a total of six hospitalizations at the Department, and the observation period (time covering all hospitalizations) amounted to 5 months and 10 days. The patient was referred to a pediatric gynecological outpatient clinic for further follow-up.

**Figure 1:**
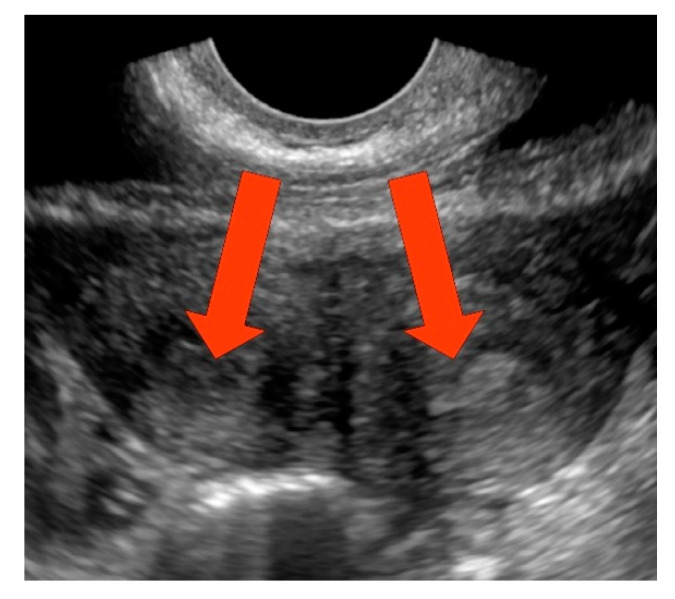
Sonogram of a 13-year-old patient imaging two uterine bodies with endometrium. There was no blood in the uterus.

A 13-year-old patient reported to the Department with painful polymenorrhoea (menstruation twice a month, every two weeks) lasting 6 months. The first menstruation occurred in the 12th year of life (1.5 years before admission to the Department), menstruation was painful since menarche, with pain both before and after the period. The patient was initially diagnosed with a double uterus at a local hospital. In the past, the patient underwent diagnostics for congenital kidney agenesis. A renoscintigraphic examination showed agenesis of the right kidney with enlargement of the left one. A compensatory mechanism was considered as one of the potential causes of this enlargement. At the Department, the patient was subject to detailed diagnostics. The cervix was examined per vaginam from the left and posterior side. From the right side, an elastic mass, which corresponded to the residual vagina, was palpated. The ultrasound revealed a reservoir measuring 71 × 40 mm, with dense content, located inferiorly to the uterus. The examination also revealed two uteri with unenlarged cavities. Diagnostic laparoscopy was subsequently performed, during which the presence of a double uterus was confirmed; moreover, no macroscopic changes were found in the left and right appendages, but the Douglas pouch was bulging into the peritoneal cavity. Subsequently, the residual vagina was incised per vaginam, draining dark, thick blood. The hole was then widened and the hemivagina was resected. No perioperative or postoperative complications occurred. Antibiotic therapy (amoxicillin and clavulanic acid) and oral estrogen-progestin therapy also commenced. Subsequent hospital admissions took place during the following months to observe the outflow of menstrual blood. At that time, no blood retention was reported in the ultrasound. During the next two hospitalizations at the Department, subsequently one and two months after the incision of the residual vagina, the right vaginal outlet was expanded under general anesthesia. During the following hospitalizations, due to the preserved patency of the residual vagina, no further expansion or recanalization procedures were performed. No blood in the uterus is shown in Figure 1.

After carrying out the diagnostic and therapeutic procedures, the patient was diagnosed with uterus didelphys with obstructed right hemivagina, right renal agenesis and hematocolpos. The patient was hospitalized at the Department for a total of eight times, and the observation period (time covering all hospitalizations) lasted 12 months and 9 days. The patient was then referred to a pediatric gynecological outpatient clinic for follow-up.

In both patients, follow-up in the outpatient clinic included history, gynecological examination and ultrasound, with particular emphasis on the patency of the hemivagina. During the observation, the patients did not report any pain that had already subsided during hospital treatment. No blood was found in the reproductive tract. In both patients, a pathological examination of the resected specimen was also done and showed no abnormalities.

## 3. Discussion

Female genital malformations arise from non-development or non-fusion of the Müllerian ducts, or failed resorption of the uterine septum [12]. Due to the occurrence of abnormalities at different stages of development, a variable morphological manifestation of defects may be observed. Although the paramesonephric tubes develop into the fallopian tubes, uterus and upper two-thirds of the vagina, anomalies of the fallopian tubes are rarely observed among Müllerian disorders. It should be emphasized that the lower third of the vagina develops from the genitourinary sinus [13]. The incidence of uterine malformations in the general female population is estimated to be at 7% to 10% [14].

**Figure 2:**
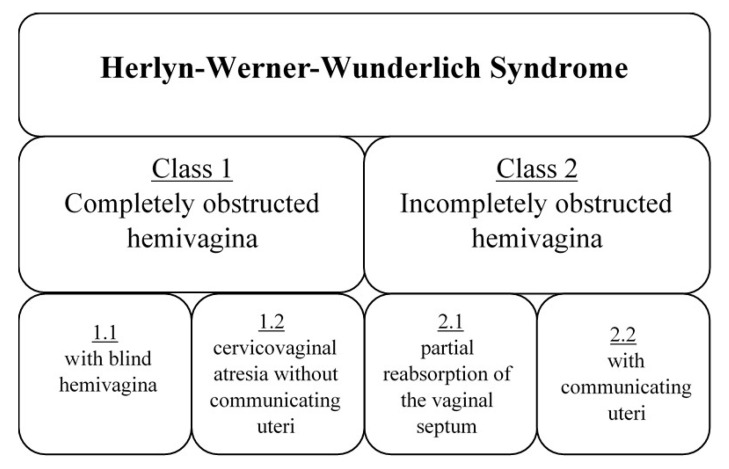
Classification of Herlyn-Werner-Wunderlich Syndrome (HWWS), based on Zhu et al. [11].

HWWS is a malformation syndrome consisting of uterus didelphys with obstructed hemivagina and ipsilateral renal agenesis. Uterus didelphys, as a component of HWWS, is included in Class 3 of the American Fertility Society classification [15]. According to European Society of Human Reproduction and Embryology (ESHRE)/European Society for Gynaecological Endoscopy (ESGE) classification, we can classify our patients as main class U3 (bicorporeal uterus), sub-class U3b (complete), co-existent classes C2 (double ‘normal’ cervix) and V2 (longitudinal obstructing vaginal septum) [16,17]. It should be noted that, morphologically, the syndrome is not uniform. Taking vaginal morphology as a criterion, HWWS is divided into Class 1 (completely obstructed hemivagina) and Class 2 (incompletely obstructed hemivagina), which in turn divide into sub-classes (Figure 2) [11].

**Figure 3:**
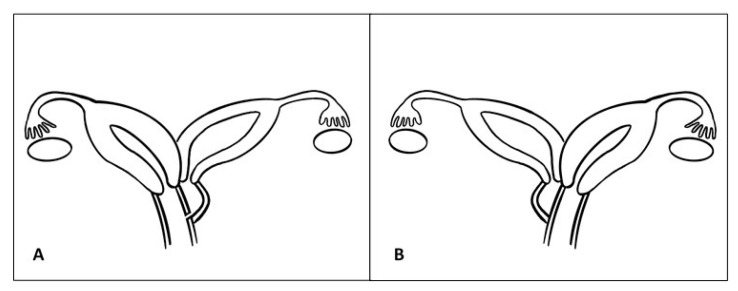
(**A**) Diagram of malformations of the 17-year-old patient. (**B**) Diagram of malformations of the 13-year-old-patient.

According to this classification, the 17-year-old patient should be qualified as sub-class 2.1, and the 13-year-old as 1.1 (Figure 3).

Clinical manifestations typically include acute or chronic lower pelvic pain occurring shortly after menarche [18], secondary to hematocolpos. However, the course of HWWS can be asymptomatic, which is associated with the normal outflow of menstrual blood through a patent collateral hemivagina. It should be noted that the presence of perforation in the obstructed left hemivagina in the 17-year-old girl, and the slight outflow of menstrual blood from the left uterine cavity, were probably the cause of a long asymptomatic period (4 years after the first menstruation). At the same time, such a long time of blood retention in the blocked vagina could contribute to the development of bacterial infection, as confirmed by microbial examination. In turn, the 13-year-old with painful polymenorrhoea, who had a completely blind hemivagina, presented with symptoms as early as 1.5 years after menarche. It should be noted that the course of the disease can result in infertility, complicated pregnancy and labor [19], as well as endometriosis. Patients with malformations are characterized by a higher prevalence of urological defects due to the common embryological origin of both systems. Improper differentiation of the mesonephric and paramesonephric ducts may be associated with renal anomalies [13]. Kidney agenesis is the most commonly described defect, although horseshoe kidney, pelvic kidney, cystic renal dysplasia, duplication of the collecting system and ectopic ureters are also found [19]. The described cases showed different methods of diagnosing kidney agenesis, which was confirmed in one case by urography and by scintigraphy in another. 

**Figure 4:**
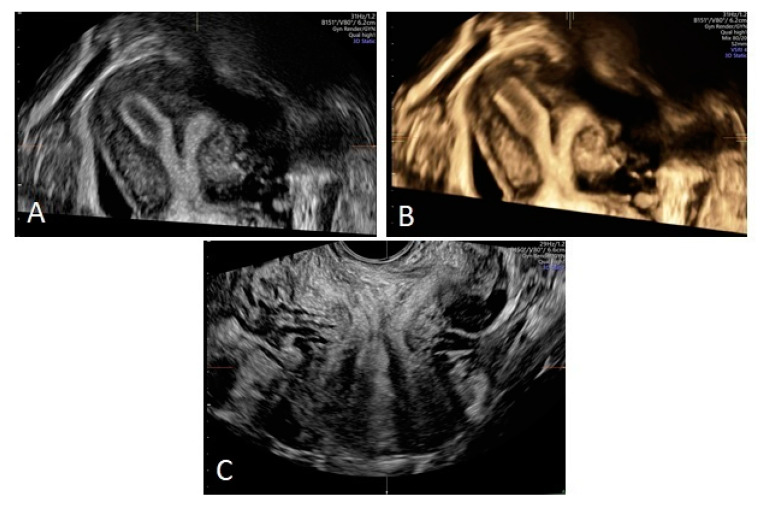
3D ultrasound of complete septate uterus. (**A**,**B**) Two uterine bodies divided by a septum; (**C**) Two uterine cervices.

Due to the normal appearance of the external genitalia, this syndrome often remains undiagnosed and asymptomatic in early childhood [20]. Diagnosis is determined on the basis of radiological examinations. Although MRI usually allows for unequivocal diagnosis of an anomaly, it is not as accessible as ultrasound, which can be performed in the A&E. Ultrasound is therefore usually helpful in establishing a diagnosis, allowing the detection of blood retention in the hemivagina and/or uterine cavity and the determination of the presence of two uterine bodies. To this extent, it aided in determining the initial diagnosis in our patients. Nowadays, 3D ultrasound is increasingly used in clinical practice. It enables the determination of the type of congenital uterine defect with high probability (Figure 4). 

It should be noted that diagnostic laparoscopy, although not mandatory, helps in establishing the diagnosis in cases where radiological imaging is inconclusive [21]. In the described cases, the combination of ultrasound and diagnostic laparoscopy allowed the determination of a definite diagnosis of the anomaly. It should be noted, however, that the diagnosis can be made without laparoscopy, and it is used in selected cases during treatment [5].

Two methods of treatment are used. According to Aveiro et al., resection of the vaginal septum is the treatment of choice for obstructed hemivagina [1], but Fascilla et al. prefer a hysteroscopic incision [5]. The advantages of the last method include, among others, avoiding the risk of the use of general anesthesia in an operating room setting and the possibility of performing it in the office [5]. Regular gynecological control is important in postoperative management, aimed at assessing the patency of the residual vagina. In some cases, it is necessary to expand the formed passage to avoid its secondary closure. Our case study showed that the treatment of HWWS is a multi-stage process that requires cooperation between the patient and a specialized treatment center. However, according to the latest information, HWWS treatment can be a one-step treatment and take place in the office [5]. Table 1 compares the diagnostic and therapeutic procedures performed in the described patients with selected cases from the literature (Table 1).

It should be noted that the topic of sexuality is often an uncomfortable topic for adolescent girls. This problem seems to be even greater in patients with congenital malformations of the genital organs associated with the procedures performed on the genital system and a sense of lack of intimacy. This can be a barrier to establishing intimate relationships. The resulting stress is further exacerbated by the fact that patients often live away from specialized treatment centers, and each hospitalization is associated with travel and the disorganization of everyday life, including education. It would therefore be helpful to include a psychologist on the clinical team [23].

## 4. Conclusions

Lower abdominal pain accompanying hematocolpos suggests HWWS as the cause of these symptoms. A 3D ultrasound can most likely recognize a congenital defect of the uterus, but inconclusive cases need to be confirmed by laparoscopy. Incising the blocked vagina and drainage of hematocolpos are the key components of treatment. The treatment of Herlyn-Werner-Wunderlich Syndrome is a multi-step process.

## Figures and Tables

**Table 1:** Comparison of the described patients with selected cases from the literature. L—left, R—right.

Patient	Age of Diagnosis	Age of Menarche	Symptoms	Examination	Obstructed Hemivagina/Renal Agenesis	Surgical Interventions
17-year-old	17 years	13 years	Acute lower abdominal pain, dysmenorrhea	Vaginal mass, pyohematocolpos, pyohematometra	L/L	Laparoscopy, vaginal septotomy, resection of vaginal septum, extension of hemivaginal entry (twice)
13-year-old	13 years	12 years	Polymenorrhoea, dysmenorrhea	Vaginal mass, hematocolpos	R/R	Laparoscopy, vaginal septotomy, resection of vaginal septum, extension of hemivaginal entry (twice)
Piccinini et al. [10]	13 years	11 years	Episodic perineal and rectal pain, abdominal fullness unrelated to eating	Abdominal mass, hydro/hemato-metrocolpos	L/L	Longitudinal vaginal septotomy, drainage of left tubo-ovarian abscess (on postoperative day 6)
Jindal et al. [12]	14 years	12 years	Recurrent pelvic pain (mainly at the time of menses), increasing lower abdominal swelling since menarche	Cystic mass arising from the pelvis, hematometro-colpos, hydrosalpinx	R/R	Vaginal septotomy, laparoscopy
Ghasemi et al. [15]	13 years	12 years	Acute abdominal pain, dysmenorrhea, fever, chills, nausea	Generalized abdominal tenderness, mild vaginal bleeding and right lateral vaginal wall bulging, cystic mass close to the right ovary, cystic mass in the right adnexa, hematocolpos, hematosalpinx, pyocolpos	R/R	Cutting the closed end of the hemilateral obstruction of the vagina, resection of vaginal septum, laparotomy (twice—during the second: appendectomy, right salpingectomy, entrolysis, abscess drainage)
Aveiro et al. [1]	13 years	13 years (5 months before)	Right lower quadrant and hypogastric pain, nausea, vomiting, dysmenorrhoea	Tenderness on right lower quadrant and hypogastric palpation, hematocolpos	R/R	Resection of the vaginal septum
Mandava et al. [22]	14 years	11 years	Acute retention of urine, fever, vomiting, lower abdominal pain	Pelvic mass palpable up to the level of the umbilicus, hematometro-colpos and hematosalpinx	L/L	Laparoscopy, resection of the vaginal septum, drainage of hematometro-colpos and hematosalpinx

## References

[1] Aveiro A.C., Miranda V., Cabral A.J., Nunes S., Paulo F., Freitas C. (2011). Herlyn-Werner-Wunderlich syndrome: A rare cause of pelvic pain in adolescent girls. BMJ Case Rep..

[2] Robbins J.B., Broadwell C., Chow L.C., Parry J.P., Sadowski E.A. (2015). Müllerian duct anomalies: Embryological development, classification, and MRI assessment. J. Magn. Reson. Imaging.

[3] Nishu D.S., Uddin M.M., Akter K., Akter S., Sarmin M., Begum S. (2019). Herlyn-Werner-Wunderlich syndrome presenting with dysmenorrhea: A case report. J. Med. Case Rep..

[4] Burgis J. (2001). Obstructive Müllerian anomalies’: Case report, diagnosis, and management. Am. J. Obstet. Gynecol..

[5] Fascilla F.D., Olivieri C., Cannone R., De Palma D., Manosperta F., Costantino A.S., Carugno J., Vicino M., Cicinelli E., Bettocchi S. (2020). In-office Hysteroscopic Treatment of Herlyn-Werner-Wunderlich Syndrome: A Case Series. J. Minim. Invasive Gynecol..

[6] Tuna T., Estevão-Costa J., Ramalho C., Fragoso A.C. (2019). Herlyn-Werner-Wunderlich Syndrome: Report of a Prenatally Recognised Case and Review of the Literature. Urology.

[7] Fachin C.G., Rocha J.L.A.S., Maltoni A.A., das Chagas Lima R.L., Zendim V.A., Agulham M.A., Tsouristakis A., dos Santos Dias A.I.B. (2019). Herlyn-Werner-Wunderlich syndrome: Diagnosis and treatment of an atypical case and review of literature. Int. J. Surg. Case Rep..

[8] Fedder J. (1990). Case Report: Uterus Didelphys Associated with Duplex Kidneys and Ureters. Acta Obstet. Gynecol. Scand..

[9] Bajaj S., Misra R., Thukral B., Gupta R. (2012). OHVIRA: Uterus didelphys, blind hemivagina and ipsilateral renal agenesis: Advantage MRI. J. Hum. Reprod. Sci..

[10] Piccinini P.S., Doski J. (2015). Herlyn-Werner-Wunderlich syndrome: A case report [Síndrome de Herlyn-Werner-Wunderlich: Relato de caso]. Rev. Bras. Ginecol. Obstet..

[11] Zhu L., Chen N., Tong J.L., Wang W., Zhang L., Lang J.H. (2015). New classification of herlyn-werner-wunderlich syndrome. Chin. Med. J..

[12] Jindal G., Kachhawa S., Meena G.L., Dhakar G. (2009). Uterus didelphys with unilateral obstructed hemivagina with hematometrocolpos and hematosalpinx with ipsilateral renal agenesis. J. Hum. Reprod. Sci..

[13] Troiano R.N. (2003). Magnetic Resonance Imaging of Mullerian Duct Anomalies of the Uterus. Top. Magn. Reson. Imaging.

[14] Acién P., Acién M., Sánchez-Ferrer M. (2004). Complex malformations of the female genital tract. New types and revision of classification. Hum. Reprod..

[15] Ghasemi M., Esmailzadeh A. (2019). An unusual appearance of the post-pubertal herlyn-werner-wunderlich syndrome with acute abdominal pain: A case report. Int. J. Reprod. Biomed..

[16] Grimbizis G.F., Gordts S., Di Spiezio Sardo A., Brucker S., De Angelis C., Gergolet M., Li T.C., Tanos V., Brölmann H., Gianaroli L. (2013). The ESHRE/ESGE consensus on the classification of female genital tract congenital anomalies. Hum. Reprod..

[17] Grimbizis G.F., Di Spiezio Sardo A., Saravelos S.H., Gordts S., Exacoustos C., Van Schoubroeck D., Bermejo C., Amso N.N., Nargund G., Timmerman D. (2016). The Thessaloniki ESHRE/ESGE consensus on diagnosis of female genital anomalies. Hum. Reprod..

[18] Zurawin R.K., Dietrich J.E., Heard M.J., Edwards C.L. (2004). Didelphic uterus and obstructed hemivagina with renal agenesis: Case report and review of the literature. J. Pediatric Adolesc. Gynecol..

[19] Shavell V.I., Montgomery S.E., Johnson S.C., Diamond M.P., Berman J.M. (2009). Complete septate uterus, obstructed hemivagina, and ipsilateral renal anomaly: Pregnancy course complicated by a rare urogenital anomaly. Arch. Gynecol. Obstet..

[20] Gupta N., Gandhi D., Gupta S., Goyal P., Li S., Kumar Y. (2018). A Variant of Herlyn-Werner-Wunderlich Syndrome Presenting with Acute Abdomen: A Case Report and Review of Literature. Glob. Pediatric Health.

[21] Smith N.A., Laufer M.R. (2007). Obstructed hemivagina and ipsilateral renal anomaly (OHVIRA) syndrome: Management and follow-up. Fertil. Steril..

[22] Mandava A., Prabhakar R.R., Smitha S. (2012). OHVIRA Syndrome (obstructed hemivagina and ipsilateral renal anomaly) with Uterus Didelphys, an Unusual Presentation. J. Pediatric Adolesc. Gynecol..

[23] Jarząbek G., Friebe Z., Szafińska A. (2004). Psychological problems affecting patients with congenital malformations of genital organs. Seksuol. Pol..

